# The Influence of Different Maternal Microbial Communities on the Development of Infant Gut and Oral Microbiota

**DOI:** 10.1038/s41598-017-09278-y

**Published:** 2017-08-30

**Authors:** Tiina Drell, Jelena Štšepetova, Jaak Simm, Kristiina Rull, Aira Aleksejeva, Anne Antson, Vallo Tillmann, Madis Metsis, Epp Sepp, Andres Salumets, Reet Mändar

**Affiliations:** 1 Competence Centre on Health Technologies, Tartu, Estonia; 2 Department of Microbiology, Institute of Biomedicine and Translational Medicine, University of Tartu, Tartu, Estonia; 3 Department of Gene Technology, Tallinn University of Technology, Tallinn, Estonia; 4 Department of Pediatrics, Institute of Clinical Medicine, University of Tartu, Tartu, Estonia; 5 Children’s Clinic of Tartu University Hospital, Tartu, Estonia; 6 Women’s Clinic of Tartu University Hospital, Tartu, Estonia; 7 Department of Obstetrics and Gynaecology, Institute of Clinical Medicine, University of Tartu, Tartu, Estonia; 8 Department of Obstetrics and Gynecology, University of Helsinki and Helsinki University Hospital, Helsinki, Finland; 9 Institute of Biomedicine and Translational Medicine, University of Tartu, Tartu, Estonia; 10 Department of Electrical Engineering (ESAT), STADIUS Center for Dynamical Systems, Signal Processing, and Data Analytics, KU Leuven, Leuven, Belgium; 11 iMinds Medical IT, Leuven, Belgium; 12 School of Natural Sciences and Health, Tallinn University, Tallinn, Estonia

## Abstract

Very few studies have analyzed how the composition of mother’s microbiota affects the development of infant’s gut and oral microbiota during the first months of life. Here, microbiota present in the mothers’ gut, vagina, breast milk, oral cavity, and mammary areola were compared with the gut and oral microbiota of their infants over the first six months following birth. Samples were collected from the aforementioned body sites from seven mothers and nine infants at three different time points over a 6-month period. Each sample was analyzed with 16S rRNA gene sequencing. The gut microbiota of the infants harbored distinct microbial communities that had low similarity with the various maternal microbiota communities. In contrast, the oral microbiota of the infants exhibited high similarity with the microbiota of the mothers’ breast milk, mammary areola and mouth. These results demonstrate that constant contact between microbial communities increases their similarity. A majority of the operational taxonomic units in infant gut and oral microbiota were also shared with the mothers’ gut and oral communities, respectively. The disparity between the similarity and the proportion of the OTUs shared between infants’ and mothers’ gut microbiota might be related to lower diversity and therefore competition in infants’ gut microbiota.

## Introduction

The composition of gut and oral microbiota develops during the first years of an infant’s life, with colonization of the gastrointestinal tract and oral cavity beginning immediately after birth. The most abundant colonizers in infant gut microbiota have been reported to include staphylococci, gammaproteobacteria (e.g., *Enterobacteriaceae*), and bifidobacteria^1, 2^. In contrast, *Streptococcus* is dominant in infant oral microbiota^3^. Accumulating evidence has shown that several maternal factors (e.g. type of delivery and feeding regimen) influence the development of the infants’ gut and oral microbiota^4, 5^. For example, immediately after birth, yet prior to removal of the *vernix caseosa*, vaginally born infants acquire bacterial communities both in their gut and oral cavity that resemble their mothers’ vaginal microbiota^6^. Similarly, infants born via caesarean section harbor communities that are similar to those found on their mothers’ skin^6^. Thus, maternal microbial communities appear to be a key source of microbes during the initial colonization process of infant gut and oral microbiota^6^. However, it has not been sufficiently characterized how different types of maternal microbial communities affect the development of infant gut and oral microbiota during the first months of life after the initial colonization process has started.

Therefore, the aim of this study was to compare the effect of different maternal microbial communities–intestinal, vaginal, oral, breast milk, and mammary areola–on the development of the infants’ gut and oral microbiota during the first six months of an infant’s life.

## Material and Methods

### Study group and sampling

Pregnant women were enrolled in this study during the first period of spontaneous onset of labor or up to 24 h before elective caesarean section with intact amniotic membranes. They were enrolled at the delivery ward of the Women’s Clinic of Tartu University Hospital between May 2012 and September 2013. All the recruited women had uncomplicated term pregnancies, they did not have any infectious diseases that required antibiotic treatment, and they had no history of diabetes or hypertensive disorders during the second half of pregnancy. A total of seven mothers (mean age: 33.1 ± 4.5 y) were recruited with their newborn babies (mother-infant pairs). Two pairs included twins, and all four infants were enrolled in this study. Only one woman was not multiparous (mean parity, 1.4 ± 0.8). Five women gave birth via caesarean section and received prophylactic cefuroxime treatment prior to the incision in the skin. All infants were term (mean gestational age, 38.9 ± 1.6 weeks) and had normal birth weights (mean weight, 3,379.3 ± 564.1 g). Clinical factors describing the mother-infant pairs are provided in Supplementary Tables S1.

**Figure 1:**
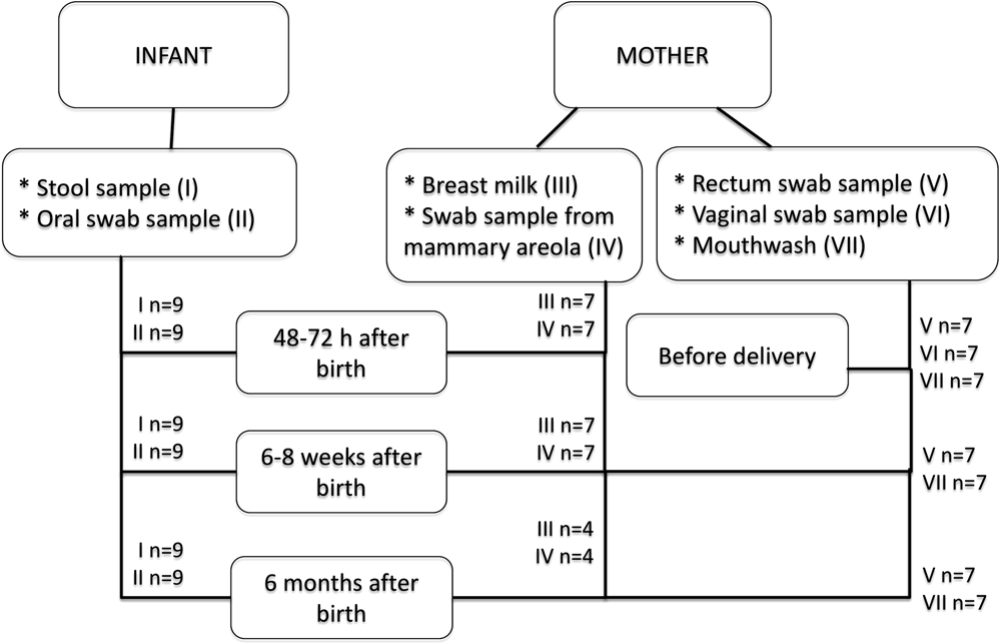
The number and type of samples that were collected from the mother-infant pairs at each of the time points indicated.

Six types of samples were collected from each mother-infant pair before birth or 48–72 h after birth, at 6–8 weeks and 6 months after birth. In addition, a vaginal swab sample was collected from each mother before giving birth. Altogether 139 samples were collected (Fig. 1). Prior to sampling, vaginal or skin disinfectant had not been used and the mammary areola had not been cleaned. All the collected samples were stored at −20 °C immediately after sampling.

Bacterioscopic smears were made from a separate vaginal swab sample that was collected from each mother before giving birth. The samples were Gram stained and scored according to the Nugent method^7^: 0–3 (normal), 4–6 (intermediate), and 7–10 (bacterial vaginosis (BV)).

### Bacterial community profiling

DNA was extracted using MoBio PowerFecal DNA Isolation Kit (Mo Bio, Carlsbad, California, USA) according to the manufacturer’s instructions. However, additional steps were included for the different types of samples. Briefly, all of the swab samples were initially vortexed for approximately 10 min in 750 μL sterile phosphate-buffered saline (PBS). 400 μL of the swab samples in PBS and 800 μL of mother’s mouthwash were added into individual dry bead tubes. 1 ml of mother’s breast milk was initially centrifuged at 6,000 rpm for 20 min. The supernatants were removed and each pellet was resuspended in 400 μL of PBS before being transferred to individual dry bead tubes. Tubes were then centrifuged at 13,200 rpm for 30 min. The supernatants were removed and the protocol for MoBio PowerFecal DNA Isolation Kit was followed from step 2. DNA from stool samples was extracted according to the manufacturer’s protocol. Samples representing the same microbial community type formed a single DNA extraction batch. Extracted DNA was stored at −20 °C prior to analysis.

Amplification of the V1–V2 hypervariable region of 16S rRNA was performed by using barcoded universal 27F-YM and 357 R primers^8^. The primer sequences were as follows: 5′-CAA GCA GAA GAC GGC ATA CGA GAT NNNNNNNN AGA GTT TGA TYM TGG CTC AG-3′ (Illumina TruSeq adapter, sample-specific 8-bp index marked as Ns followed by 27F-YM at the 3′ end) and 5′-AAT GAT ACG GCG ACC ACC GAG ATC TAC ACC TGC TGC CTY CCG TA-3′ (TruSeq universal adapter sequence and 357 R at the 3′ end). The conditions for amplification included: 10 min at 98 °C, followed by 5 cycles of 30 s at 98 °C, 30 s at 55 °C, and 45 s at 72 °C, 30 cycles of 30 s at 98 °C and 60 s at 72 °C, and a final extension step at 72 °C for 10 min.

PCR reactions had a total volume of 20 μL, with 10 μL of Phusion High-Fidelity PCR Master Mix (Thermo Scientific, Waltham, Massachusetts, USA), 5 μL of DNA template, and each primer at the concentration of 0.2 μM. PCR products were purified using Agencourt AMPure XP (Beckman Coulter, Brea, California, USA) and were sequenced with an Illumina MiSeq system at the Genome Centre in University of Tartu, Estonia (single end sequencing using MiSeq v2 kit and 300 cycles).

Analysis of bifidobacteria with DGGE and real-time PCR was performed by using the methodology described in Supplementary methods.

### Data and statistical analysis

MOTHUR software 1.27.0 was used to trim, denoise and align the sequences obtained to generate operational taxonomic units (OTUs) and to assign taxonomy. Sequences were trimmed and discarded based on a quality score <25 and length >225 bp, respectively. OTUs were generated by using an average neighbor hierarchical clustering algorithm with the identity threshold of 97%. Reference sequences for aligned 16S rRNA gene sequences were obtained from the SILVA ribosomal RNA database and comparisons with taxonomic assignments were performed with a Ńaive Bayesian classifier with a confidence cutoff of 90%. OTUs with less than two sequences and OTUs present in fewer than two samples were also discarded. To determine the most likely species name for pivotal OTUs mapped to genus *Lactobacillus*, an additional taxonomic assignment against the NCBI 16S ribosomal RNA sequences database was performed with BLASTN.

Statistical analyses were performed with R 3.4.0 software. The cut-off values of the minimal number of trimmed sequences assigned to the samples were set to individual community types after which the sequence counts were normalized for the whole dataset. To analyze general bacterial diversity and similarity between the samples, Shannon diversity index, Cosine similarity index (CSI) and Jaccard distance values were calculated, respectively. CSI measures the similarity between samples taking into account both the abundance and the prevalence of the OTUs (on the scale of 0 to 1 with 1 being the most similar) when Jaccard distance, which is a measure of dissimilarity, indicates to the differences between samples based only on the prevalence of the OTUs (on the scale of 0 to 1 with 1 being the most dissimilar). Continuous variables were compared with the Wilcoxon rank-sum test. The probability of an OTU present in infants’ gut or oral microbiota being shared with a specific maternal community type was analyzed using logistic regression analysis. A factor representing the presence (or absence) of an OTU in both infant’s and mother’s community type was a dependent variable whereas the identifier for the pairs of samples (infant’s stool or oral sample paired with mother’s rectum, vaginal, breast milk, mammary areola or mouthwash sample) was a predictor. Only the dominant OTUs colonizing infants’ gut and oral microbiota (relative abundance >0.005) were analyzed with separate logistic regression analyses.

All analyses were carried out with the Holm-Bonferroni correction. P-values less than 0.05 were considered to be statistically significant.

### Ethical considerations

This study received approval from the Research Ethics Committee of the University of Tartu (no. 210T-7) and written informed consent was obtained from each participant upon admission to the delivery ward. All methods were performed in accordance with the relevant guidelines and regulations.

## Results

### Bacterial community profiling using 16S rRNA sequencing

A total of 1,106,448 high quality sequence reads were generated in this study. The cut-off set for the minimal number of trimmed sequences assigned to the samples varied from 200 to 1,400 depending on the community type (Supplementary Table S2). At these cut-off values the rarefaction curves for at least 90% of the samples reached a 5% plateau. In total, 135 samples and 1,530 OTUs exceeded these cut-off values and were further analyzed (GenBank accession numbers for representative sequences of the OTUs: KP117311-KP118840). One breast milk, one mother’s oral and two infants’ oral samples did not cross these cut-off values. The retrieved OTUs were distributed among 12 phyla, 89 families, and 170 genera.

The highest Shannon diversity index values were observed in the mothers’ gut and oral microbiota and these communities differed significantly from the infants’ gut and oral microbiota that harbored significantly lower microbial diversity (p < 0.001). The diversity of the microbial communities did not change significantly during the analyzed time period (Supplementary Figure S1).

### Similarity between infants’ gut and oral microbiota versus mothers’ microbial communities from various sites

**Figure 2:**
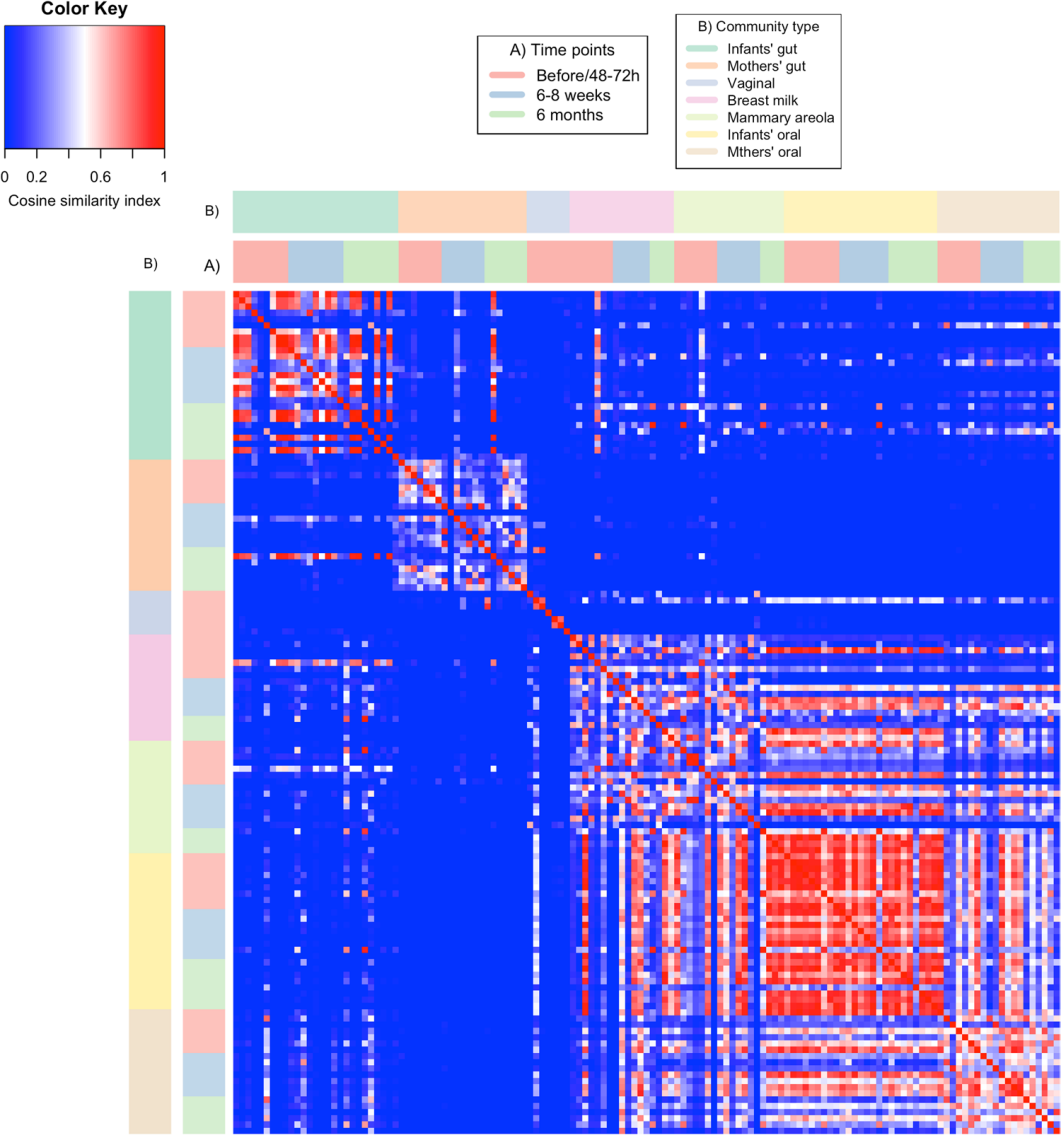
Correlation plot representing the Cosine similarity index values between the analyzed microbial community types. The time points represent the sampling times in relation to birth of the infants.

**Figure 3:**
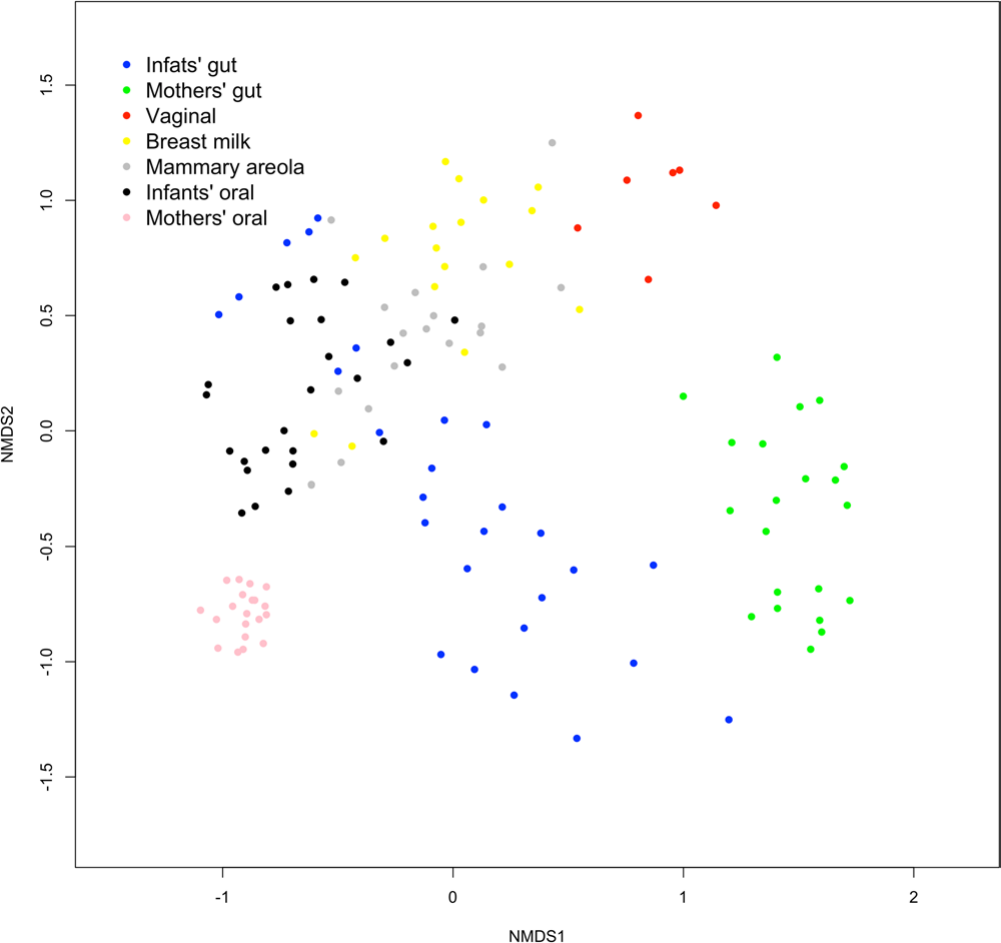
Non-metric multidimensional scaling (NMDS) plot visualizing the Jaccard distance between the samples of analyzed microbial community types.

**Figure 4:**
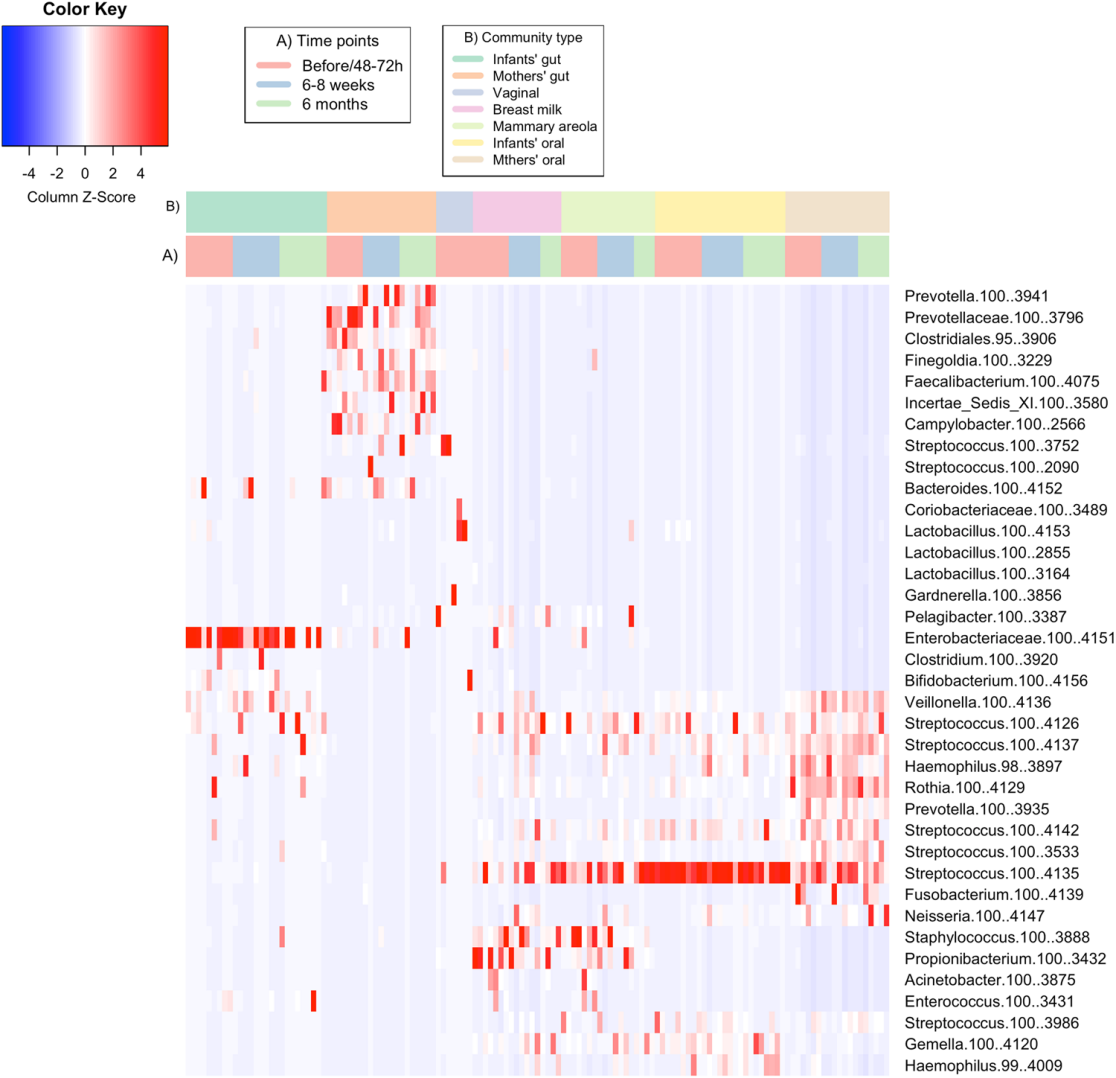
Relative abundance of the most dominant OTUs that colonized the analyzed community types.

Based on the Cosine similarity index and Jaccard distance values, distinct patterns of similarity and dissimilarity were observed among the samples examined (Figs 2 and 3). These patterns were also concordant with the observed distribution of dominant OTUs between the community types (Fig. 4).

The gut microbiota of the infants did not share similarities with any of the analyzed maternal community types throughout the study, yet high similarity was observed between the infants’ oral microbiota and the microbiota colonizing the mothers’ breast milk, the mammary areola, and the oral cavity (Fig. 2). These four community types harbored a combination of dominant OTUs belonging mostly to genus *Streptococcus* that were present in each population with similarly high relative abundance (Fig. 4). Exceptions were the OTUs that mapped to the genera *Staphylococcus* and *Propionibacterium*. These OTUs had low relative abundance (<0.01) in both the infants’ and mothers’ oral microbiota.

**Figure 5:**
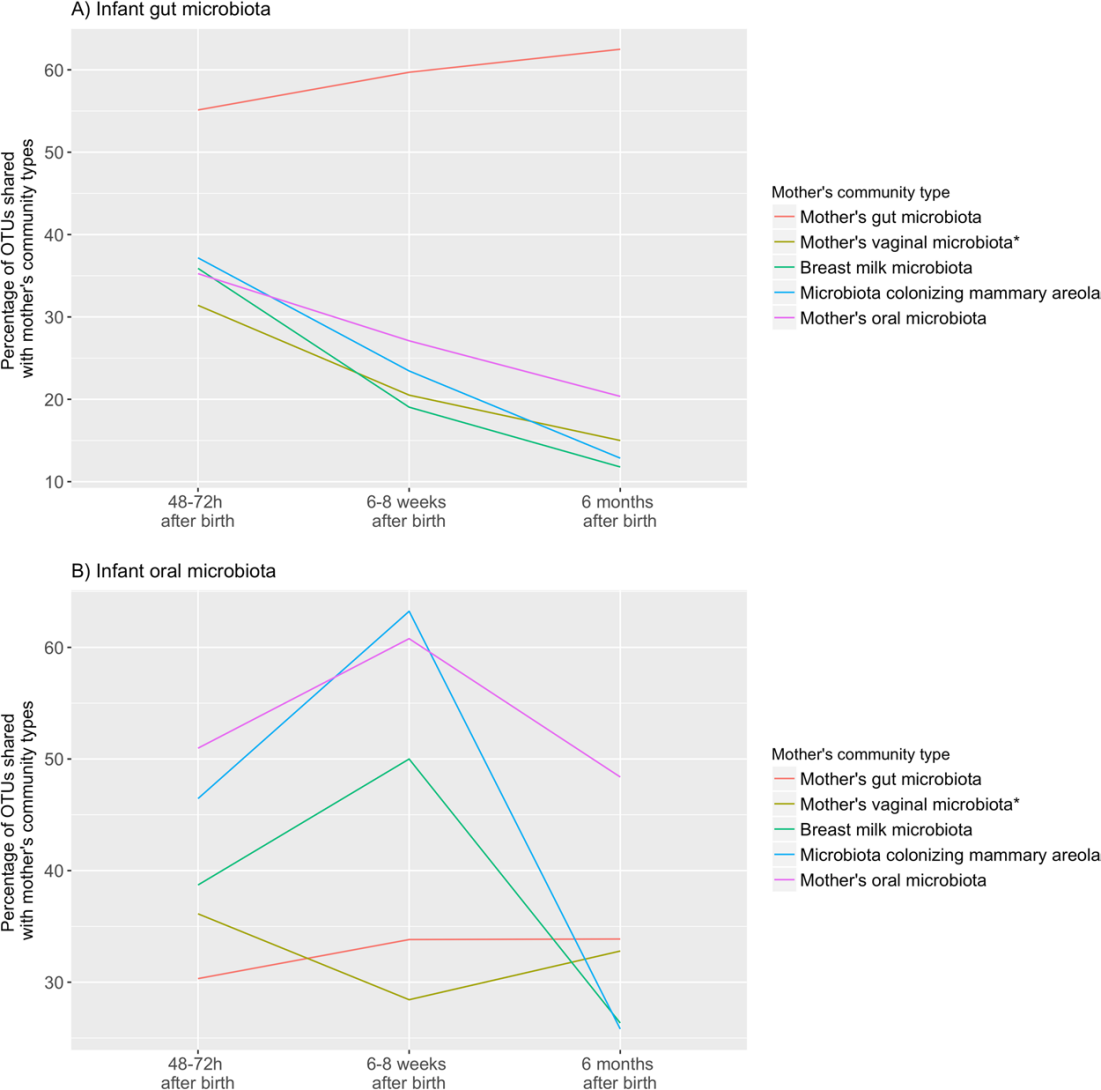
Proportion of OTUs observed in the pooled data of infants’ gut (**A**) and oral (**B**) microbiota that were shared with mothers’ community types. *Infants’ gut and oral microbiota at every analyzed time point were compared to vaginal microbiota observed in mothers before giving birth.

**Figure 6:**
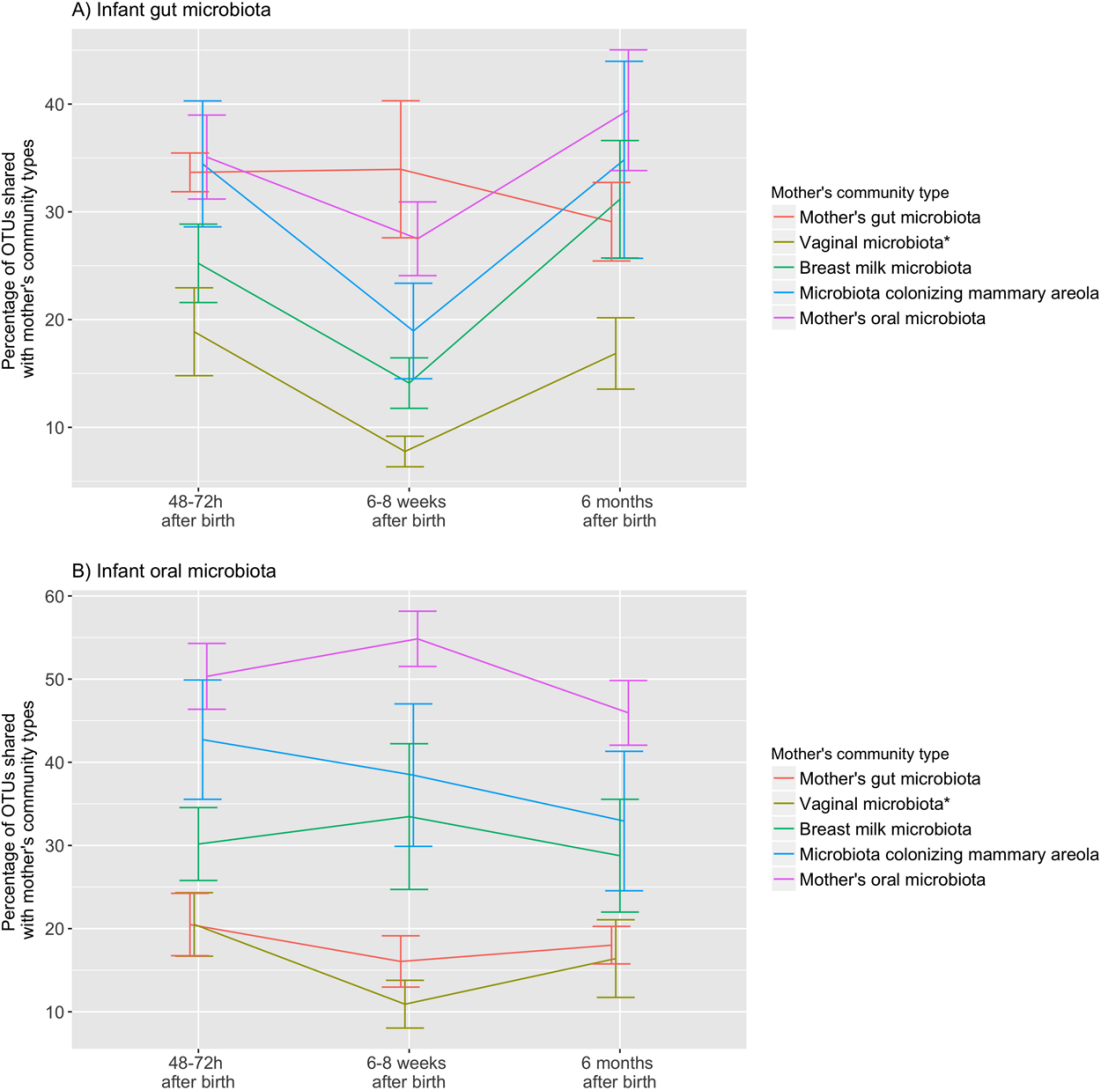
Average proportion with 95% confidence interval of OTUs observed in infants’ gut (**A**) and oral (**B**) microbiota that were shared with his or her mother’s community types (the proportion shared between specific mother-infant pairs). *Infants’ gut and oral microbiota at every analyzed time point was compared to vaginal microbiota observed in mothers before giving birth.

Most of the OTUs observed in the infants’ gut and oral microbiota were simultaneously shared with several of the maternal community types and we did not observe significantly higher probability of the OTUs dominating infants’ gut and oral microbiota being shared with a specific maternal community type over other maternal community types. Only 20%, 26% and 28% of the OTUs colonizing the infants’ gut, and 22%, 19% and 27% colonizing infants’ oral microbiota at 48–72 h, 6–8 weeks and 6 months after birth, respectively, were observed exclusively in these community types. All of these OTUs had very low relative abundance (<0.01). Highest proportion of the OTUs observed in the pooled data of infants’ gut microbiota (55%, 60% and 63%, respectively at 48–72 h, 6–8 weeks and 6 months after birth) were observed in the pooled data of mothers’ gut microbiota (mainly members of *Clostridiales* and *Bacteroidales*, which represented more than 68% of the OTUs observed in both communities) (Fig. 5), but when analyzing individual mother-infant pairs, the proportion of the OTUs shared between an infant’s and his or her own mother’s gut microbiota was not that high (mean [SD] proportion of the OTUs shared between mother-infant pairs: 32% [13%], 34% [19%] and 29% [11%], respectively at 48–72 h, 6–8 weeks and 6 months after birth). This proportion was similar to the proportion shared between infant’s gut microbiota and the communities colonizing his or her mother’s oral cavity (35% [12%], 28% [10%] and 39% [15%], respectively at 48–72 h, 6–8 weeks and 6 months after birth) and mammary areola (34% [18%], 19% [13%] and 35% [18%], respectively at 48–72 h, 6–8 weeks and 6 months after birth) (Fig. 6). In the infants’ oral microbiota, the highest proportion of OTUs was shared with the mothers’ oral microbiota (members of various taxa) when analyzed both between two community types (i.e. all samples pooled together) (51%, 61% and 48%, respectively at 48–72 h, 6–8 weeks and 6 months after birth; Fig. 5) and between individual mother-infant pairs (mean [SD] proportion of the OTUs 50% [12%], 55% [9%] and 46% [10%], respectively at 48–72 h, 6–8 weeks and 6 months after birth; Fig. 6).

**Figure 7:**
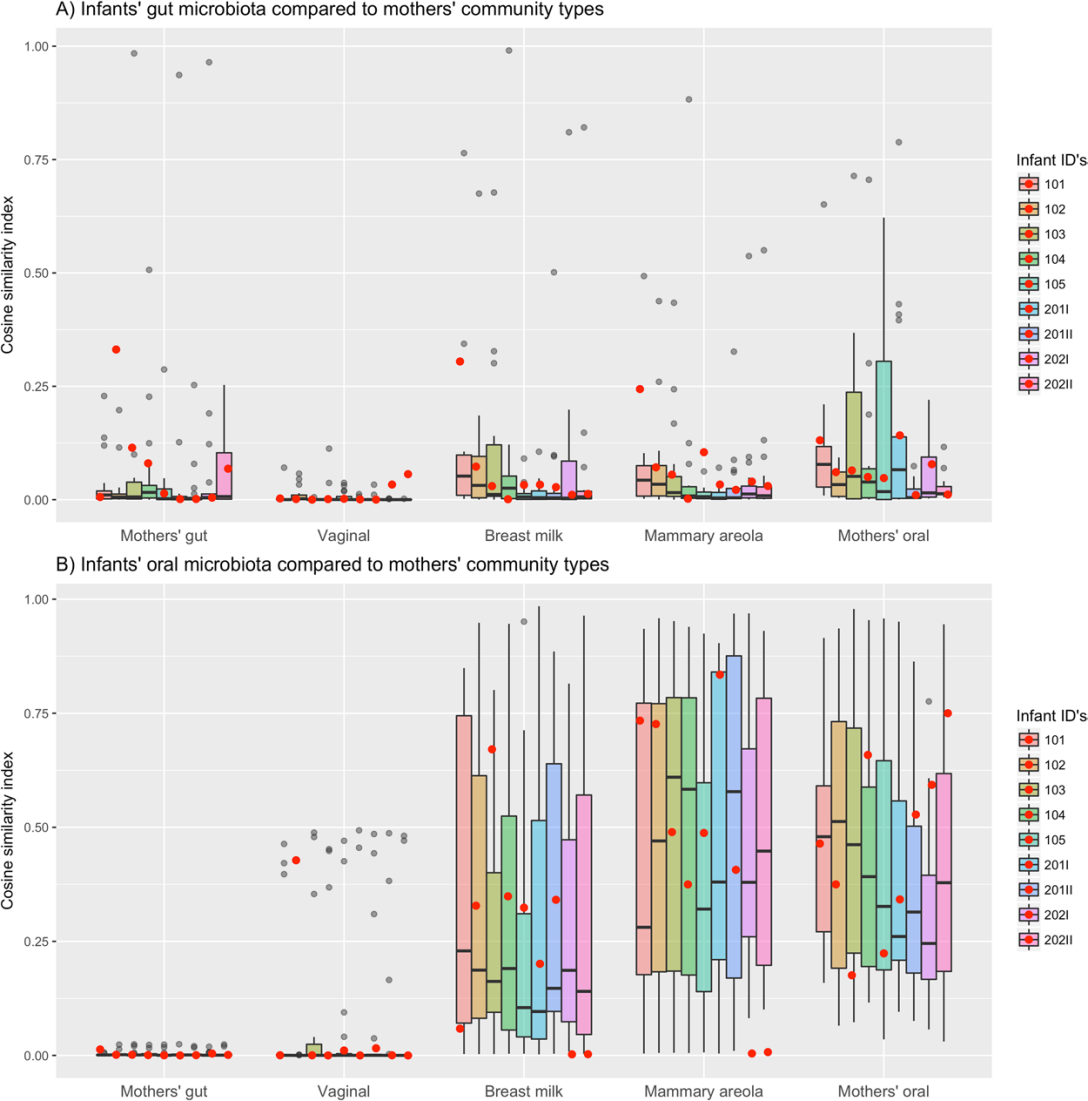
Average Cosine similarity index between infant’s gut (**A**) and oral (**B**) microbiota and their own mother’s microbial community types (red dots), and the community types observed in the rest of the mothers (Tukey boxplot).

The infants’ gut and oral microbiota did not exhibit significantly greater similarity to their own mother’s microbial communities than to the other mothers’ microbial communities and the similarity did not change significantly during the study (Fig. 7 and Supplementary Figure S2).

### The composition of the infants’ gut and oral microbiota

*Firmicutes* and *Proteobacteria* were equally dominant in the infants’ gut microbiota throughout the study (mean relative abundance [SD] was 0.42 [0.27] vs. 0.39 [0.3], respectively) (Supplementary Figure S3). On lower taxonomic levels, *Enterobacteriaceae* from phylum *Proteobacteria* dominated (0.37 [0.31]), while *Clostridiales* (mostly *Lachnospiraceae*)*, Streptococcaceae*, and *Veillonellaceae* from phylum *Firmicutes* were also abundant (0.13 [0.2], 0.12 [0.24], and 0.1 [0.15], respectively). In contrast, *Bacteroidaceae* and *Bifidobacteriaceae* were relatively scarce (0.06 [0.14] and 0.05 [0.08], respectively). Real-time PCR detected high levels of bifidobacteria in the infants’ gut microbiota throughout the study (average [SD]: 5,974 [15,208] copies per mg of feces; Supplementary Figure S4), and no significant fluctuations were observed. There were six bifidobacterial taxa observed in the infants’ gut microbiota (*B. longum, B. longum subsp. infantis*, *B. longum subsp. longum, B. breve, B. pseudocatenulatum* and *B. adolescentis*), with an average of 3.6^2^ bifidobacterial taxa colonized per infant. The highest number of bifidobacterial taxa was observed in the twin pairs, with six taxa identified in ID-s 201I and 201II and five taxa identified in ID-s 202I and 202II. Gut microbiota was dynamic in most of the infants over the time period studied (Supplementary Figure S5-A). The composition was moderately similar among infants and it did not change significantly during the study period when considering both the CSI and Jaccard distance.

*Firmicutes* was also prominent in the infants’ oral microbiota throughout the study (mean [SD] relative abundance, 0.82 [0.16]) (Supplementary Figure S3). On lower taxonomic levels, *Streptococcaceae* dominated (0.7 [0.18]), followed by *Pasteurellaceae* (0.08 [0.09]). The infants’ oral microbiota was relatively stable during the time period studied (Supplementary Figure S6-C) and was very similar among the infants.

The gut and oral microbiota of the twins in this study did not exhibit a greater similarity among these paired siblings than was observed among the other infants (mean [SD] CSI: 0.35 [0.38] vs. 0.32 [0.34] and 0.76 [0.24] vs. 0.75 [0.22] for the gut and oral microbiota among twins vs. the rest of the infants, respectively). Also, gut and oral microbiota of infants born vaginally *versus* via caesarean section did not exhibit greater similarity (0.22 [0.16] vs. 0.35 [0.38] and 0.8 [0.12] vs. 0.73 [0.24] for the gut and oral microbiota among infants born vaginally vs. via caesarean section, respectively).

### The composition of microbial communities colonizing the mothers

*Firmicutes* dominated all the maternal community types analysed, but *Bacteroidetes* and *Actinobacteria* were also very abundant in the gut and vaginal microbiota, respectively (Supplementary Figure S3). On lower taxonomic levels, the communities differed substantially with gut microbiota being dominated by order *Clostridiales* (0.4 [0.14]) along with family *Prevotellaceae* (0.2 [0.14]); oral microbiota by *Streptococcaceae* (0.4 [0.18]), followed by *Prevotellaceae, Micrococcaceae, Fusobacteriaceae*, *Pasteurellaceae*, and *Veillonellaceae* (0.09 [0.05], 0.09 [0.07], 0.08 [0.12], 0.08 [0.06], and 0.06 [0.03], respectively); breast milk microbiota and the microbiota colonizing mammary areola by *Streptococcus, Staphylococcus*, *Propionibacterium, Gemella, Acinetobacter*, and *Enterococcus* in both instances (0.32 [0.28], 0.12 [0.17], 0.09 [0.1], 0.05 [0.08], 0.03 [0.06] and 0.01 [0.04]; 0.48 [0.33], 0.13 [0.17], 0.05 [0.09], 0.05 [0.08], 0.03 [0.09] and 0.02 [0.05], respectively in each case). Gut and oral microbiota was relatively stable and generally similar between different women over the time period studied (Supplementary Figures S5-B and S6-D). In most of the women, the composition of breast milk microbiota and the microbiota colonizing mammary areola was dynamic during the time period studied (Supplementary Figures S6-A and S6-B). All the women had normal to intermediate Nugent scores (Supplementary Table S3) and the composition of vaginal microbiota varied among women (Supplementary Figure S7).

## Discussion

To our knowledge this is one of the first studies to analyze the effect of mothers’ microbiota of various body sites to infants’ gut and oral microbiota. Based on the mother-infant pairs that we examined, infant gut microbiota appears to harbor a distinctive microbial community that exhibits low similarity with the microbiota that colonize the mother’s gut, vaginal, skin, breast milk, and oral cavity during the first six months of the infant’s life. In contrast, the infants’ oral microbiota, as well as the mothers’ breast milk microbiota, mammary areola microbiota, and oral microbiota exhibited high similarity to each other.

Although approximately 63% of the OTUs observed in an infant’s gut microbiota were also observed in the mother’s gut microbiota (Fig. 5), only on average 32% were shared between individual mother-infant pairs, which was not considerably higher than the proportion of OTUs shared with the mother’s oral and skin communities (Fig. 6). High numbers of OTUs observed in both infants’ and mothers’ gut microbiota that had considerably different relative abundances (Fig. 4) may be related to the lower species diversity (Supplementary Figure S1) and therefore competition in infants’ gut microbiota as hypothesized by Asnicar *et al*.^9^. Relatively low levels of OTUs shared between infant’s and one’s own mother’s gut microbiota and considerable numbers of OTUs shared between the infant’s gut microbiota and microbiota colonizing one’s own mother’s oral cavity and mammary areola may on the other hand be related to the delivery mode of the infants participating in this study as most of them were born via caesarean section (Supplementary Table S1-B). Bäckhed *et al*.^10^ have shown that 72% of the early colonizers of the vaginally delivered infants’ gut matches species found in the stool of their own mother, whereas only 41% of these species are detected in infants born via caesarean section. They also observed enriched presence of bacteria typically know to be of skin and mouth origin colonizing the gut microbiota of C-section infants^10^.

The infants’ oral microbiota shared a high similarity with communities colonizing oral cavity, breast milk and mammary areola of the mothers (Fig. 2). Although the same OTUs dominated in all four aforementioned community types (Fig. 4), dominance of the same OTUs mapped to genus *Streptococcus* in infants’ and mothers’ oral microbiota indicates that mother’s oral microbiota has the biggest influence on the development of infants’ oral microbiota during the first six months of life, because members of *Streptococcus* are the predominant habitants of oral microbiota in both infants and adults^3^. Additionally, the highest number of OTUs colonizing the infants’ oral microbiota was observed in the mothers’ oral microbiota and this was also the case when analyzing the proportion of shared OTUs between individual mother-infant pairs (Figs 5 and 6). The similarity with oral microbiota may be the result of maternal habits of infant care (e.g., frequent use of the same spoon, licking the pacifier, kissing on the mouth). While not all mother-to-infant contacts involve direct interactions of oral microbiota, a similar observation was made in a recent study where the salivary microbiota of romantically involved partners exhibited increasing similarity when partners kissed at relatively high frequencies^11^. Mother’s oral microbiota may also influence the development of an infant’s oral microbiota via the placenta. For example, in a recent study by Aagaard *et al*.^12^ mothers’ oral microbes appeared to be present in the placenta^12^.

High abundance of *Streptococcus* OTUs observed in communities colonizing breast milk and mammary areola and the abundant presence of *Staphylococcus* and *Propionibacterium*, which are typical colonizers of human skin^13^, in breast milk microbiota (Supplementary Figure S6) indicates that there may be a retrograde reflux taking place during breastfeeding as have been hypothesized by Ansicar *et al*.^9^. Because mammary areolae were not cleaned before sampling the study may be overestimating the similarity between infants’ oral microbiota and microbiota colonizing mammary areola.

We did not observe higher similarity of microbial communities between infants and their own mothers when compared to the other mothers (Fig. 7). This may seem surprising considering that several previous studies have observed mother-to-infant transmission of bacterial strains^9, 10^, but most probably our results indicate to the shortcoming of 16S rRNA gene sequencing in identifying taxa on lower than genus level^14^. Thus, our results do not rule out significant similarities between individual mother-infant pairs (and distinctively different community pattern from other mothers) when analysing the composition of the communities on strain level.

Another limitation of this study was the small sample size, which is most probably the reason why we did not observe the effect of type of delivery and feeding regimen to the community composition of infants’ gut and oral microbiota. These effects are well described by many of the previous studies [e.g. refs 6, 10]. Nevertheless, the highest number of bifidobacterial taxa was observed among twins’ gut microbiota who were breastfed for a shorter period of time and received prebiotic formula instead. This result is consistent with a study conducted by Barrett *et al*.^15^ where the greatest number of bifidobacterial strains and diversity were observed in the infants who received formula containing prebiotics (e.g. galacto-oligosaccharides and polyfructose)^15^.

In conclusion, the infants’ gut microbiota was found to be dissimilar from all of the maternal community types which were analyzed in this study while the infants’ oral microbiota exhibited a high similarity with the mothers’ oral, breast milk, and mammary areola microbiota. These results emphasize the significant effect of constant contact between these microbial communities. However, both the infant gut and oral microbiota were found to share the highest proportion of the OTUs with the corresponding maternal community type. The disparity between the similarity and the proportion of the OTUs observed both in the infants’ and mothers’ gut microbiota might be related to lower species diversity and therefore lower competition in infants’ gut microbiota, which leads to difference in the abundance of the shared OTUs.

## Electronic supplementary material

Supplementary Info
