# Supplementary material for: The Influence of Different Maternal Microbial Communities on the Development of Infant Gut and Oral Microbiota
Source: Sci Rep. 2017 Aug 30;7:9940. doi: 10.1038/s41598-017-09278-y (PMC5577157; doi:10.1038/s41598-017-09278-y)

THE INFLUENCE OF DIFFERENT MATERNAL MICROBIAL COMMUNITIES ON THE DEVELOPMENT OF INFANT GUT AND ORAL MICROBIOTA

Tiina Drell*[a, b, c], Jelena Štšepetova[a], Jaak Simm[b, j, k], Kristiina Rull[f, g], Aira Aleksejeva[f], Anne Antson[d, e], Vallo Tillmann[d, e], Madis Metsis[l], Epp Sepp[a], Andres Salumets[c, g, h, i], Reet Mändar[a, c]

a) Department of Microbiology, Institute of Biomedicine and Translational Medicine, University of Tartu, Tartu, Estonia

b) Department of Gene Technology, Tallinn University of Technology, Tallinn, Estonia

c) Competence Centre on Health Technologies, Tartu, Estonia

d) Department of Pediatrics, Institute of Clinical Medicine, University of Tartu, Tartu, Estonia

e) Children's Clinic of Tartu University Hospital, Tartu, Estonia

f) Women's Clinic of Tartu University Hospital, Tartu, Estonia

g) Department of Obstetrics and Gynaecology, Institute of Clinical Medicine, University of Tartu, Tartu, Estonia

h) Department of Obstetrics and Gynecology, University of Helsinki and Helsinki University Hospital, Helsinki, Finland

i) Institute of Biomedicine and Translational Medicine, University of Tartu, Tartu, Estonia

j) Department of Electrical Engineering (ESAT), STADIUS Center for Dynamical Systems, Signal Processing, and Data Analytics, KU Leuven, Leuven, Belgium

k) iMinds Medical IT, Leuven, Belgium

l) School of Natural Sciences and Health, Tallinn University, Tallinn, Estonia

Supplementary Tables S1. Clinical factors describing the mother-infant pairs analyzed.

A) Information about the mother

Factors	ID 101	ID 102	ID 103	ID 104	ID 105	ID 201[1]	ID 202[1]
**Age (y)**	34	32	41	31	35	26	33
**No. of children (excl. participating infants)**	2	1	1	2	2	0	2
**Type of previous delivery(ies)**	Caesarian (both)	Caesarian	Vaginal	Vaginal (both)	Caesarian (both)	NA	Vaginal (both)
**Body mass index (BMI) before pregnancy**	21.8	22.1	No data	23.9	46.9	27.7	21.3
**BMI WHO classification**	Normal	Normal	No data	Normal	Obese class III	Overweight	Normal
**Regular menstrual cycle**	Yes	Yes	No	Yes	Yes	Yes	Yes
**Cycle length (days)**	28	26	No data	28	28	28	27
**First period after delivery during the study period**	0	0	13th week	No data	No data	30th week	6th week
**Common cold symptoms during pregnancy[2]**	20th week	25th week	20th week	No symptoms	34th week	No symptoms	21st week
**Genital symptoms during the study period +treatment**	Vaginal discharge	Vulvovaginal candidiasis (incl. inching and lesions) + topical clotrimazole & systemic econazole treatment at 36th week during pregnancy.	No symptoms	No symptoms	No symptoms	No symptoms	No symptoms

		Topical clotrimazole treatment during weeks 2–6 after delivery.					
**Other inflammatory symptoms after delivery+ treatment**	No symptoms	No symptoms	Lactation mastitis at 13th week	No symptoms	No symptoms	Inflammation of caesarean section incision + cefuroxime treatment at 1st week	Lactation mastitis at 6th week
**Allergies**	No	Yes (dust, pollen, cats)	No	Yes (unspecified)	No	No	No
**Frequency of washing breasts during the study period**	Daily	Less than daily	Daily	Daily	Less than daily	Daily	Daily
**Washing hands before holding infant**	In most cases	In most cases	In most cases	Rarely	Always	In most cases	In most cases
**Washing hands after changing diapers**	Rarely	In most cases	In most cases	Rarely	Always	Always	In most cases
**Smoking prior to pregnancy**	No	No	Yes	Yes	No	Yes	No
**Smoking during pregnancy[3]**	No	No	Yes	No	No	No	No

1 The mother-infant pairs indicated as ID 201 and ID 202 included twin siblings. ID 201 had dizygotic and ID 202 monozygotic twins.

2 None of the mothers were treated with antibiotics.

3 None of the mothers smoked after delivery during the study period.

B) Information about the delivery and newborn(s)

Factors	ID 101	ID 102	ID 103	ID 104	ID 105	ID 201[1]	ID 202[1]
Type of delivery	Caesarian[4]	Vaginal	Caesarian[4]	Vaginal	Caesarian[4]	Caesarian[4]	Caesarian[4]
Reason for caesarian delivery	Uterine scarring		No data		Uterine scarring	One embryo in lower uterine segment	Uterus bicornis
Time between rupture of membranes and birth (min)	No rupture	37	No rupture	119	7	No rupture	No rupture
Gestational age (week)	40	40	41	38	39	37	37
Gender of the infant(s)	Female	Male	Male	Female	Male	Female (I)<br>Female (II)	Female (I)<br>Male (II)
Birth weight (g)	3276	4372	3272	3684	4112	2836 (I)<br>2770 (II)	2946 (I)<br>3146 (II)
Birth height (cm)	51	53	50	50	49	48 (I)<br>46 (II)	46 (I)<br>50 (II)
Weight at 8 weeks (g)	5390	6470	5060	5410	6000	4710 (I)<br>4080 (II)	No data
Height at 8 weeks (cm)	59	62	56	55	58	57 (I)<br>53 (II)	No data
Weight at 6 months (g)	8500	9780	No data	No data	7450	7550 (I)<br>6630 (II)	7840 (I)<br>9940 (II)
Height at 6 months (cm)	69	72	No data	No data	67	71 (I)<br>66 (II)	64 (I)<br>76 (II)
Duration of breastfeeding	Throughout the study period	19 weeks	23 weeks	No data	No data	13 weeks	12 weeks

**Start of formula feeding**	No data	From 72h after birth[5]	No data	No data	No data	Immediately after birth[5]	Immediately after birth[5]
**Introduction to weaning**	21st week	19th week	23rd week	No data	18th week	17th week	20th week
**Symptoms observed during the study period + treatment**	No symptoms	Coryza (9th week and 16th week)	Conjuctivitis (3rd week) Oral thrush (4th week) + topical chloramphenicol treatment (eye drops)	No data	Cough (9th week)	I: Coryza (6th week), diarrhea (23rd week) II: Coryza (6th week), cough (6th week), diarrhea (23rd week)	I: Cough and coryza (4th week), coryza (20th week) II: Cough and coryza (2nd week), oral trush (8th week), coryza (20th week)

1 Mother-infant pairs indicated as ID 201 and ID 202 included twin siblings. ID 201 had dizygotic twins and ID 202 had monozygotic twins.

4 All caesarian deliveries were elective.

5 Infants received formula containing prebiotics (galactooligosaccharides and polyfructose) prior to weaning.

Supplementary methods. Analysis of bifidobacteria with denaturing gradient gel electrophoresis (DGGE) and real-time PCR

1) **Primers and probes**

DGGE, sequencing of DGGE amplicons and real-time PCR were performed with the primers and probes listed in Table 1. Primers and probes used for both methods targeted the 16S rRNA gene. The oligonucleotide probe used in real-time PCR for the detection of the genus Bifidobacterium was labeled with a 5' reporter dye, (VIC), and a 3' quencher, (NFQ-MGB), respectively and the probe used for the detection of the total count of bacteria was labelled a 5' reporter dye, (FAM), and a 3' quencher, (TAMRA), respectively (Applied Biosystems, Foster City, California, USA).

Table 1. Primers and probes used in the study

**Method**	**Primers (5'-3')**	**References**
DGGE	Im3: CGGGTGCTICCCACTTTCATG<br>Im26: GATTCTGGCTCAGGATGAACG<br>Bif164: GATTTAGGTGACACTATAG<br>Bif662-f: CCACCGTTACACCGGGAA<br>Bif662-r+GC: CGCCCGCCGCGCGGCGGGCGGGGCGGGGGCACGGGGGGCCACCGTTACACCGGGAA	Satokari et al., 2001*
Sequencing of DGGE amplicons	SP6: TAATACGACTCACTATAGG<br>T7: GTGAAGCTTACGGT(C/T)TACCTTGTTACGACTT	Promega
Real-time PCR	Bif-f: GGGATGCTGGTGTGGAAGAGA<br>Bif-r: TGCTCGCGTCCACTATCCAGT<br>Bif(Probe): (VIC)-TCAAACCACCACGCGCCA-(NFQ-MGB)<br>Eub-f: TCCTACGGGAGGCAGCAGT	Haarman et al., 2007**

	Eub-r: GGACTACCAGGGTATCTAATCCTGTT<br>Eub(probe): FAM-CGTATTACCGCGGCTGCTGGCAC-TAMRA	

* Satokari RM, Vaughan EE, Akkermans ADL, Saarela M, de Vos WM. (2001). Bifidobacterial diversity in human feces detected by genus-specific PCR and denaturing gradient gel electrophoresis. Appl Environ Microbiol 67:504–513.

** Haarman M, Knol J (2005). Quantitative Real-Time PCR assays to identify and quantify fecal Bifidobacterium species in infants receiving a prebiotic infant formula. Appl Environ Microbiol 71:2318–2324.

2) PCR-DGGE

PCR was performed in a reaction volume of 50µl containing 25 µl Maxima Hot Start PCR Master Mix (Thermo Scientific, Waltham, Massachusetts, USA), 200 ng of DNA solution and primers at a concentration of 10 µM. The DGGE cycling parameters were 5 min at 94 °C, followed by 35 cycles of 30 s at 94 °C, 30 s at 62 °C (for primers Bif164 and Bif662) or 30 s at 57ºC (for primer Im-3 and Im-26), and a final extension at 72 °C for 10 min and 30 s.

DGGE analysis of PCR amplicons was performed with the DcodeTM System (Bio-Rad, Hercules, California, USA). Polyacrylamide gels (8% [wt/vol] acrylamide-bisacrylamide [37.5:1]) in 0.5X Tris-acetic acid-EDTA buffers with a denaturing gradient were prepared with a gradient mixer and EconoPump (Bio-Rad). The gradients ranged from 45 to 60% to provide separation of the amplicons.

3) Sequencing of DGGE amplicons

Cloning of the PCR products

PCR amplicons (Bif164-r-Bif662-f) were purified and concentrated with a QIAquick PCR purification kit (Qiagen, Hilden, Germany) according to the manufacturer's instructions. Purified amplicons were then cloned into *E.coli* JM109 using the pGEM-T vector system (Promega, Madison, Wisconsin, USA). Colonies of ampicillin-resistant transformants were randomly picked from each sample and were subjected to PCR with the pGEM-T-specific primers T7 and SP6 (Table 1) from the lyzed cells to check the size of the inserts. Plasmid DNA of selected transformants was isolated using a QIAprep Spin Miniprep Kit (Qiagen).

Sequencing

Sequencing reactions were performed with a BigDye Terminator CA v3.1 Cycle Sequencing kit (Applied Biosystem) according to the manufacturer's instructions. The sequences obtained were analyzed using an automatic LI-COR DNA Sequencer 4000L (Licor, Lincoln, Nebraska, USA) and were corrected manually. All of the sequences were thereafter identified using BLASTN and the NCBI nucleotide database.

4) Real-time PCR

Plasmids construction and standards for qRT-PCR

In order to establish quantitative assays, plasmid standards were generated using the method described by Bartosch *et al*. (2004)*. Briefly, the amplified 16S rRNA gene region (amplified with primers Bif-f and Bif-r) of *B. bifidum* DSM 20456 was cloned into chemically competent *E. coli* JM109 cells using the pGEM-T Easy vector system (Promega, Madison, USA). Plasmids were purified with NucleoSpin PlasmidQuick pure Kit according to the manufacturer's instructions (Macherey-Nagel, Germany). The purified plasmids were quantified using spectrophotometry (NanoDrop ND-1000, Thermo Scientific). Quantification of the target DNA was achieved by using serial tenfold dilution from 10^5 to 10^1 plasmid copies of the previously quantified plasmid standards.

* Bartosch S, Fite A, Macfarlane GT, McMurdo ET. (2004). Characterization of Bacterial Communities in Feces from Healthy Elderly Volunteers and Hospitalized Elderly Patients by Using Real-Time PCR and Effects of Antibiotic Treatment on the Fecal Microbiota. Appl Environ Microbiol 6:3575-3581

Real-time PCR

Multiplex quantitative real-time PCR was performed with a 7500 Fast Real-Time PCR System (Thermo Scientific) using optical-grade 96-well plates. PCR reactions had a total volume of 25 µL which included 12.5 µL of TaqMan® Universal PCR Master Mix (Thermo Scientific), 2 µL of DNA template, 400 nM (Bif-f and Bif-r primers) and 800nM (Eub-f and Eub-r primers) of corresponding forward and reverse primers and 100

nM (Bif) and 200nM (Eub) of probes. Cycling parameters were 2 min at 50 °C and 10 min at 95 °C for an initial denaturation step, followed by 40 cycles of 15 s at 95 °C and 1 min at 60 °C. Data from triplicate samples were analyzed using Sequence Detection Software version 1.6.3 (Thermo Scientific).

Supplementary Table S2. The cut-off value for the minimal number of trimmed sequences assigned to the samples in different community types where the rarefaction curves for at least 90% of the samples of these community types reached a 5% plateau.

**Community type**	**The cut-off set for the minimal number of trimmed sequences assigned to the samples**
Infant's gut	300
Mother's gut	1400
Vaginal	1000
Breast milk	400
Mammary areola	600
Infant's oral	200
Mother's oral	900

Supplementary Figure S1. The Tukey boxplot representing Shannon diversity index in analyzed community types.

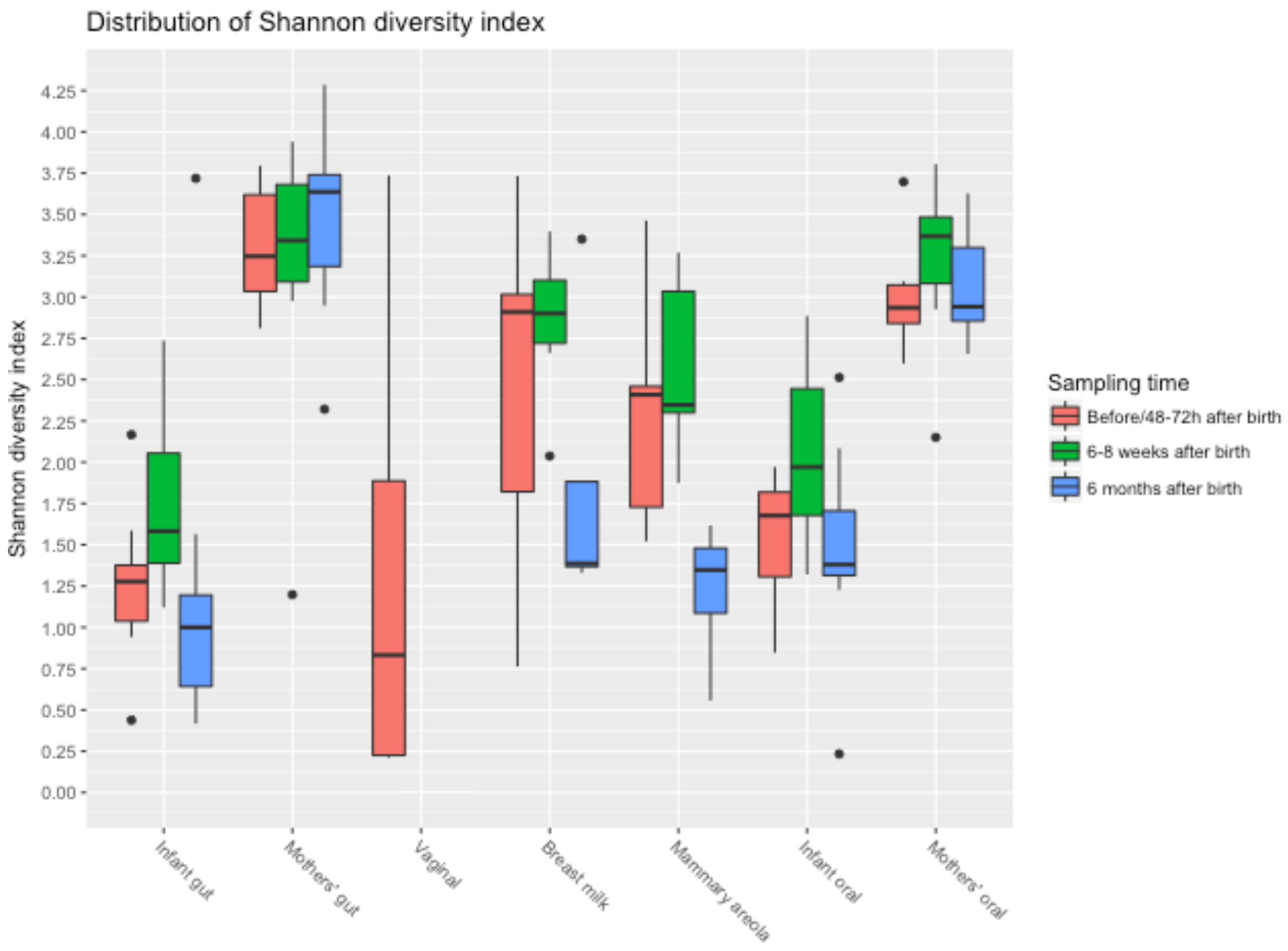

Supplementary Figure S2. Average Jaccard distance between infant's gut (A) and oral (B) microbiota and their own mother's microbial community types (red dots), and the community types observed in the rest of the mothers (Tukey boxplot).

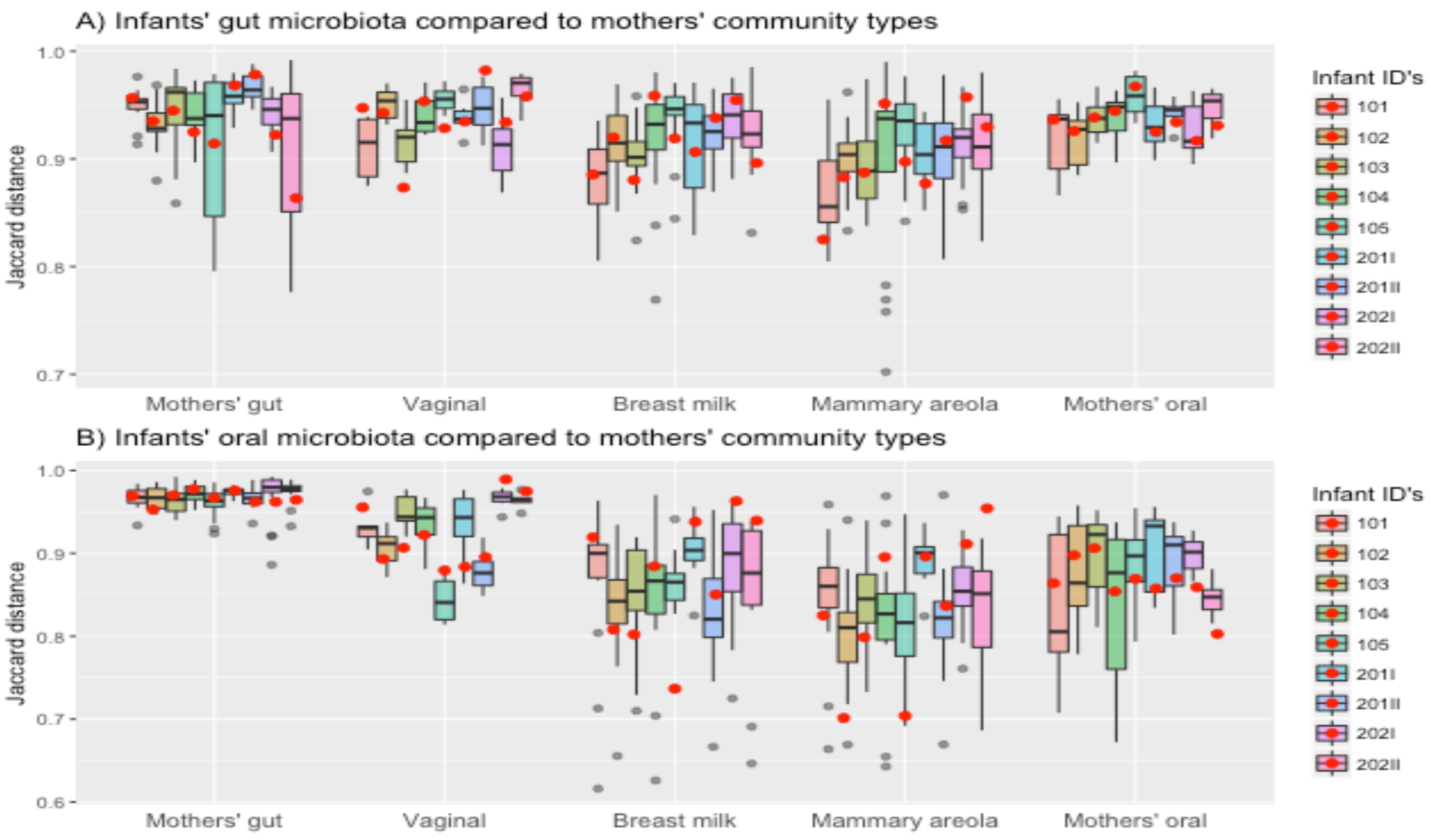

Supplementary Figure S3. Distribution of phyla.

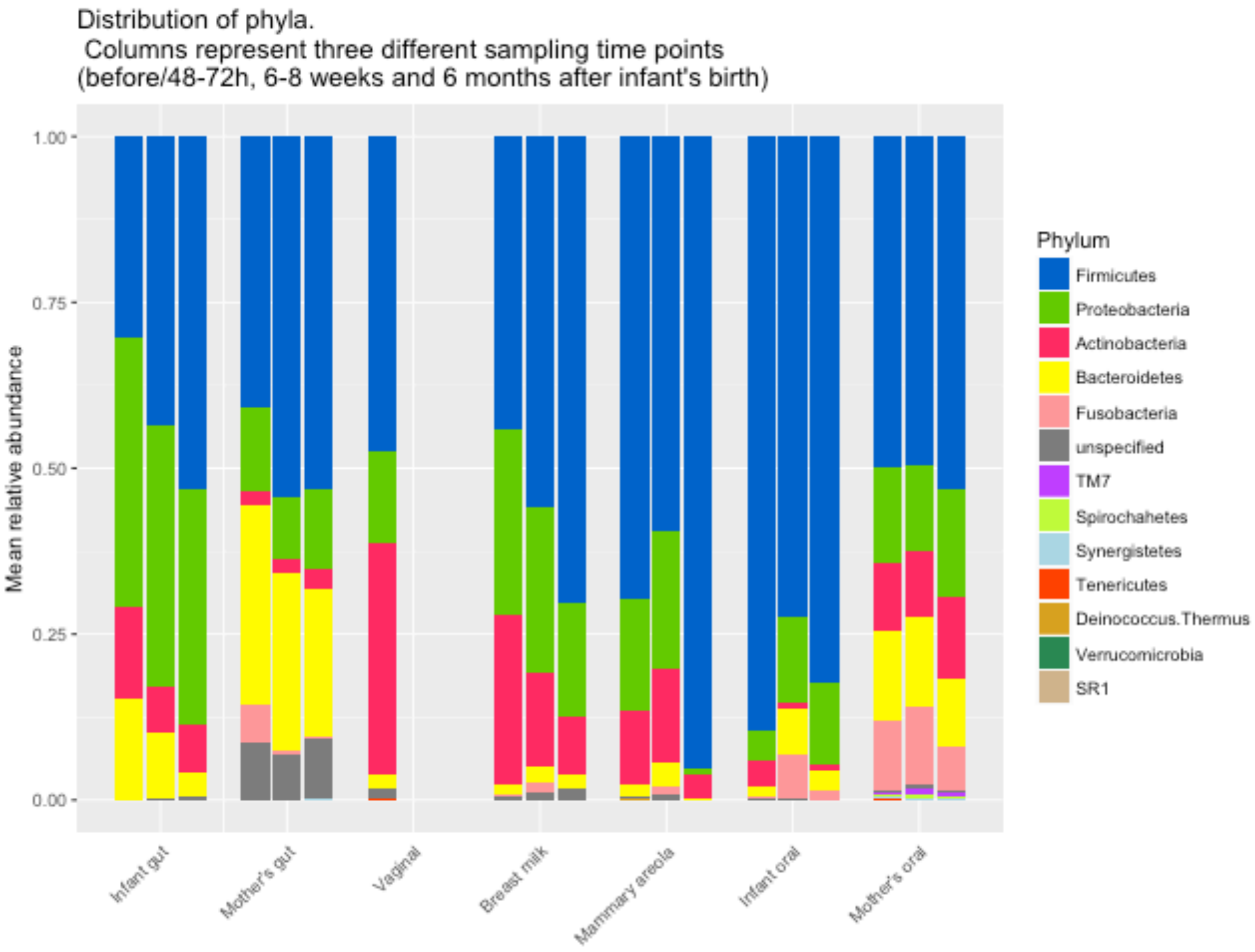

Supplementary Table S3. Nugent scores determined for the analyzed vaginal samples collected from each mother before giving birth. Each morphotype (*Lactobacillus* [LB], *Gardnerella* [Gv], and *Mobiluncus* [Mob]) was evaluated semi-quantitatively: 0 (no morphotypes in the visible area); 1+ (0–1 morphotypes in the visible area); 2+ (1–4 morphotypes in the visible area); 3+ (5–30 morphotypes in the visible area); 4+ (> 30 morphotypes in the visible area).

The Nugent score results for each morphotype were combined to provide a final score as follows: 0–3 (normal), 4–6 (intermediate), and 7–10 (bacterial vaginosis (BV)).

**ID**	**Time points**	**LB**	**Gv**	**Mob**	**Nugent score**	**Clue cells**	**White blood cells**	**Other observations**	**Estimated value**
101	1	2	4	0	**6**	-	-	-	Intermediate
102	1	0	0	0	**0**	-	Low	-	Normal
103	1	0	0	0	**0**	-	Low	-	Normal
104	1	0	4	1	**5**	-	Low	-	Intermediate
105	1	1	4	0	**5**	-	Low	-	Intermediate
201	1	0	2	0	**2**	-	Low	-	Normal
202	1	0	1	0	1	-	Moderate	-	Normal

Supplementary Figure S4. Distribution and abundance of Bifidobacteria.

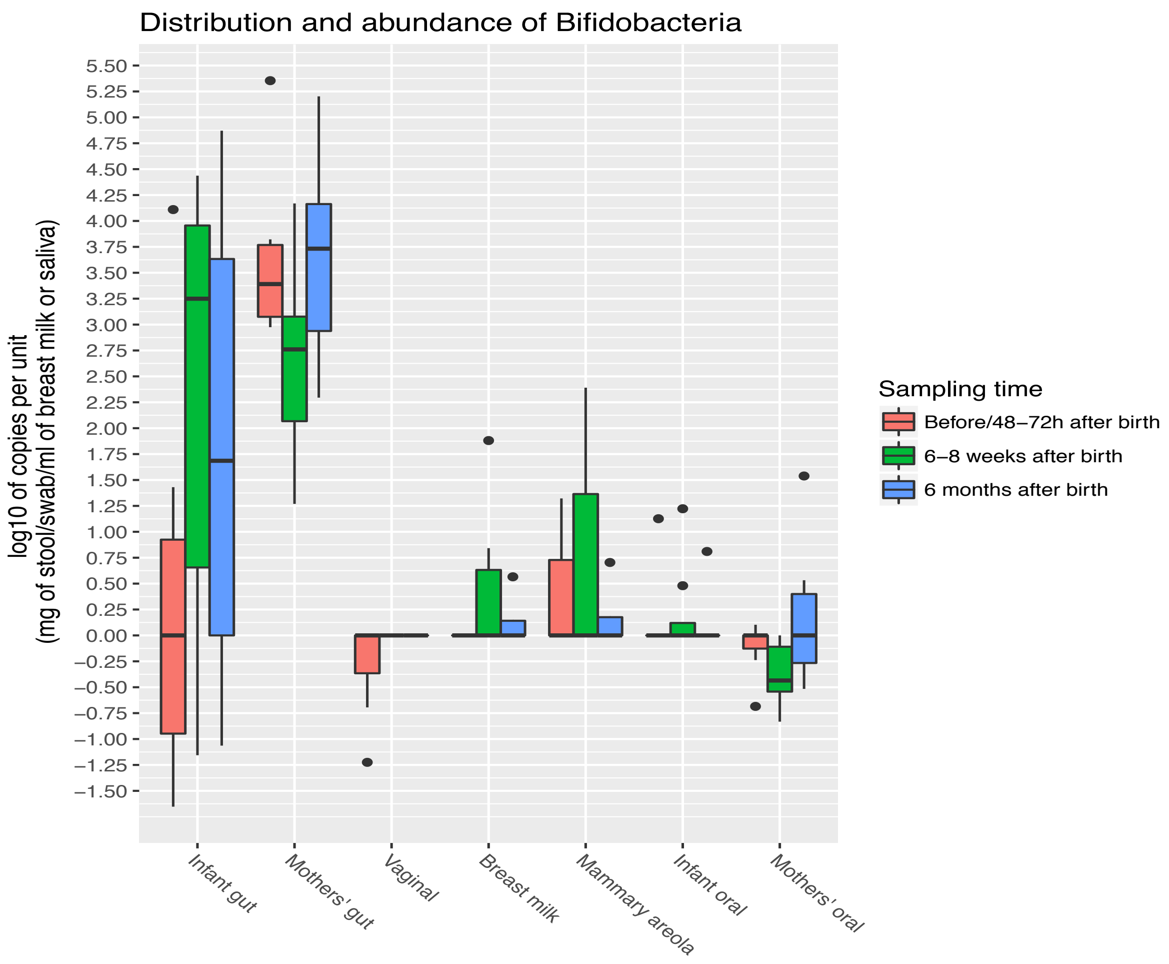

Supplementary Figure S5. Dominating taxa in the infants' (A) and mothers' (B) gut microbiota. The columns represent the three different time points for sample collection for each individual (ID) as follows: collection of a rectal swab sample from the mothers immediately before giving birth; the collection of infant stool samples 48–72 h after birth, and then the collection of both samples at 6–8 weeks after birth and at 6 months after birth.

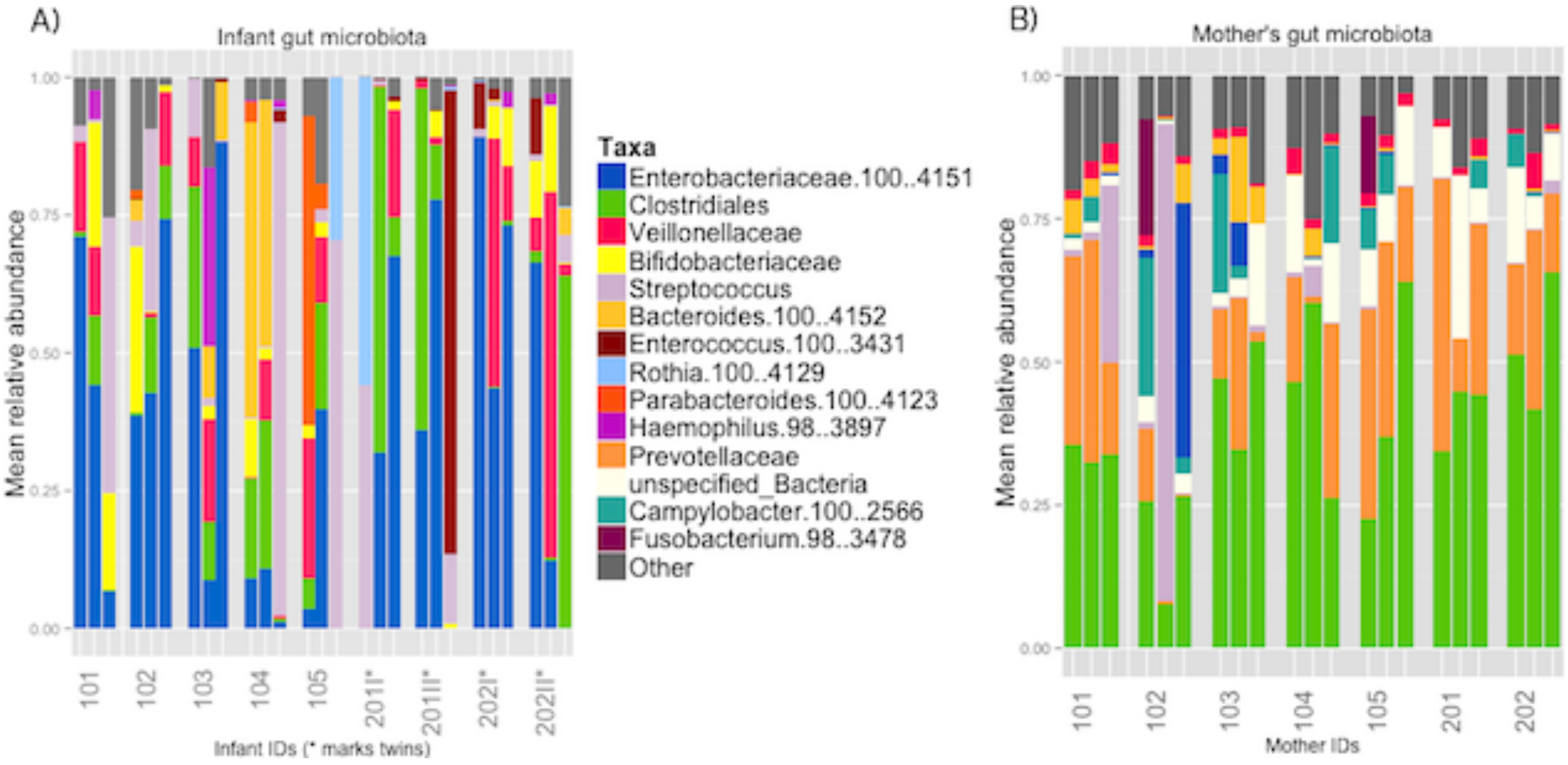

Supplementary Figure S6. Dominating taxa in the breast milk (A), mammary areola (B), infant's oral (C), and mother's oral (D) microbiota. The columns represent the different time points for sample collection from each individual (ID) which included: collection of the mothers' mouthwash sample just prior to giving birth; collection of samples 48–72 h after birth (of the breast milk, mammary areola, and infants' oral swab samples); collection of samples 6–8 weeks after birth (values are missing for ID 202's breast milk and ID 201I's infant oral sample); and collection of samples 6 months after birth (values are missing for IDs 104, 201, and 202 for breast milk and mammary areola samples. ID 202's mouthwash sample and ID 202II's infant's oral sample).

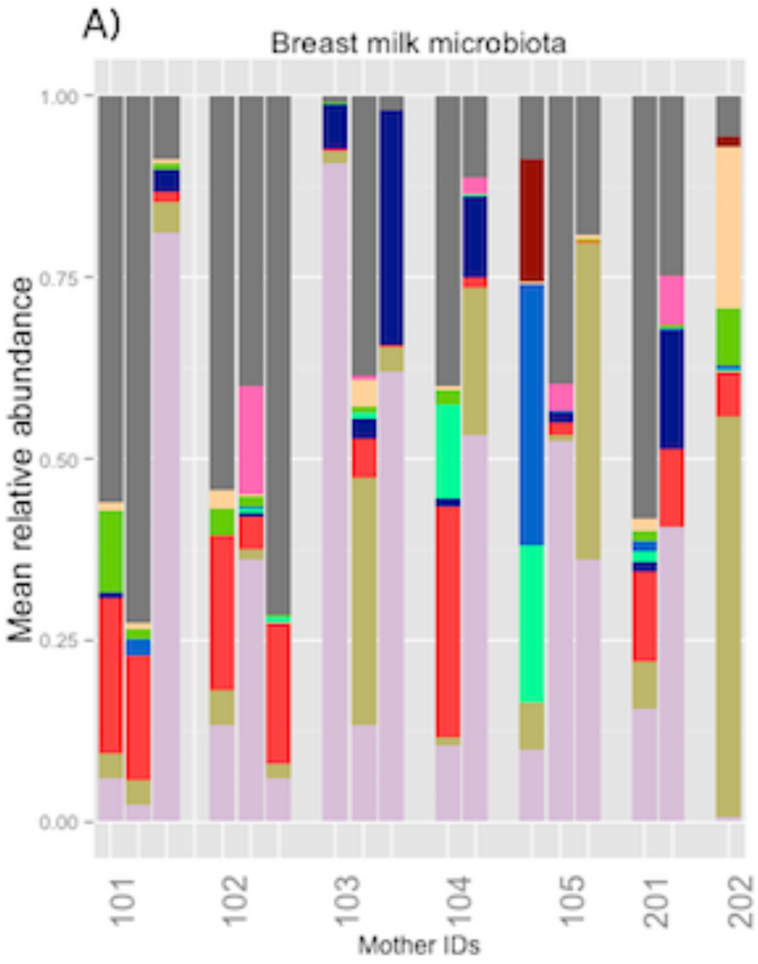

A)
Breast milk microbiota
Mean relative abundance
1.00
0.75
0.50
0.25
0.00
101
102
103
104
105
201
202
Mother IDs
B)
Microbiota colonizing mammary areola
Mean relative abundance
1.00
0.75
0.50
0.25
0.00
101
102
103
104
105
201
202
Mother IDs

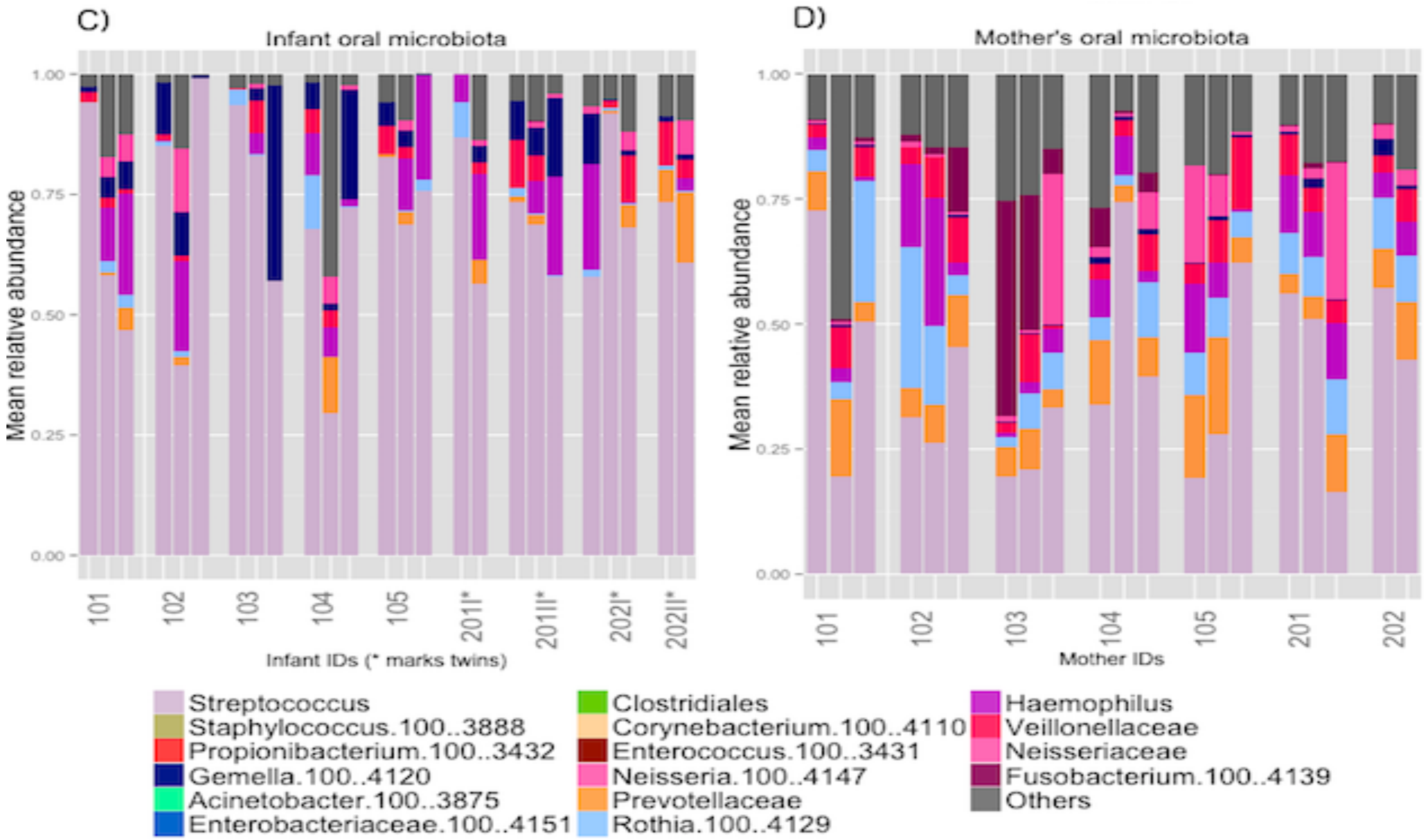
C)
Infant oral microbiota
Mean relative abundance
1.00
0.75
0.50
0.25
0.00
101
102
103
104
105
201I*
201II*
202I*
202II*
Infant IDs (* marks twins)
D)
Mother's oral microbiota
Mean relative abundance
1.00
0.75
0.50
0.25
0.00
101
102
103
104
105
201
202
Mother IDs
Streptococcus
Staphylococcus.100..3888
Propionibacterium.100..3432
Gemella.100..4120
Acinetobacter.100..3875
Enterobacteriaceae.100..4151
Clostridiales
Corynebacterium.100..4110
Enterococcus.100..3431
Neisseria.100..4147
Prevotellaceae
Rothia.100..4129
Haemophilus
Veillonellaceae
Neisseriaceae
Fusobacterium.100..4139
Others

Supplementary Figure S7. Dominating taxa in the mothers' vaginal microbiota.

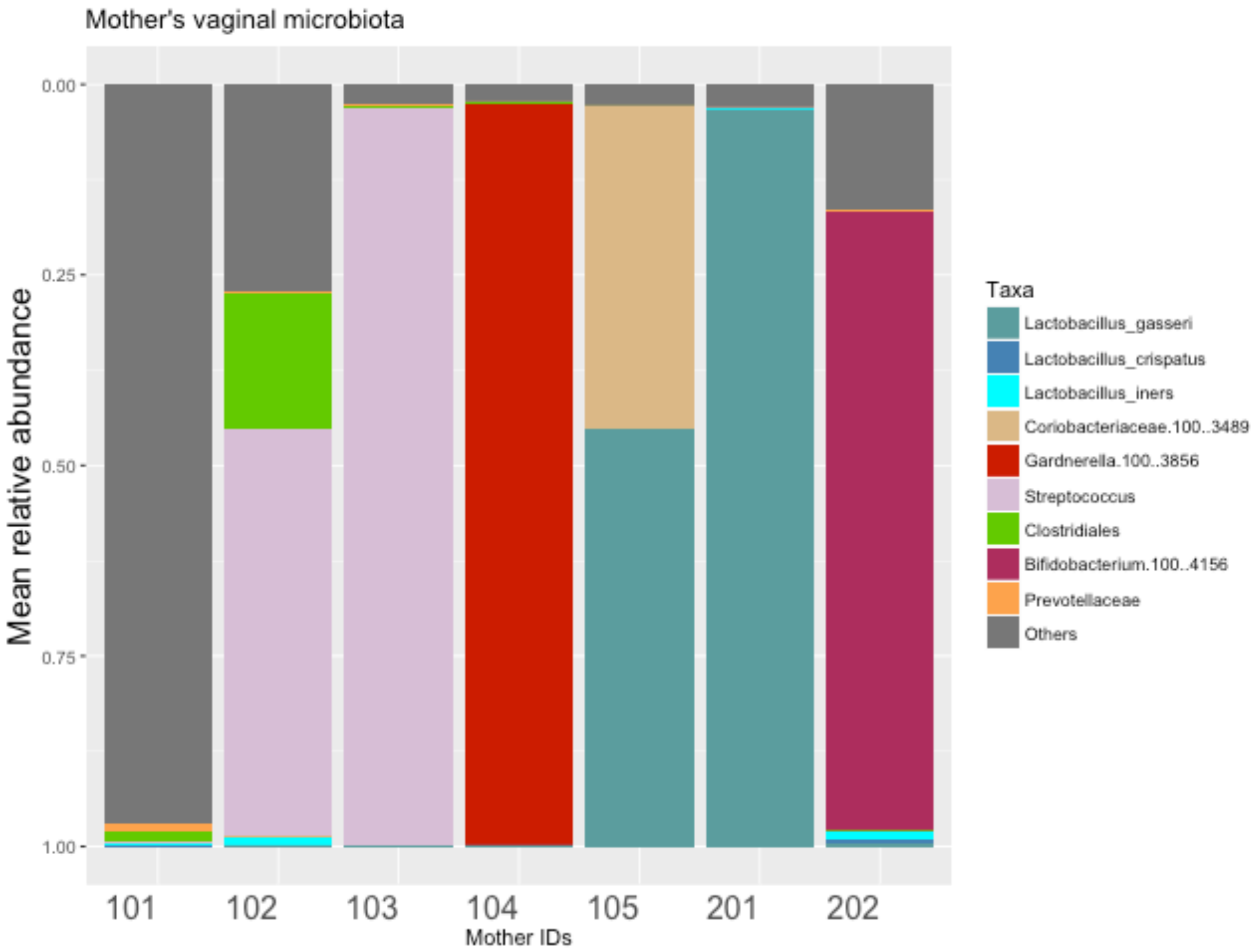

Supplement: Supplementary file 1 — Supplementary Info [file 41598_2017_9278_MOESM1_ESM.pdf]
